# Supplementary material for: Real-Time 3D Imaging and Inhibition Analysis of Various Amyloid Aggregations Using Quantum Dots
Source: Int J Mol Sci. 2020 Mar 13;21(6):1978. doi: 10.3390/ijms21061978 (PMC7139405; doi:10.3390/ijms21061978)
Supplement: Supplementary file 1 [file ijms-21-01978-s001.pdf]

Supplemental Materials
Supplemental Figures

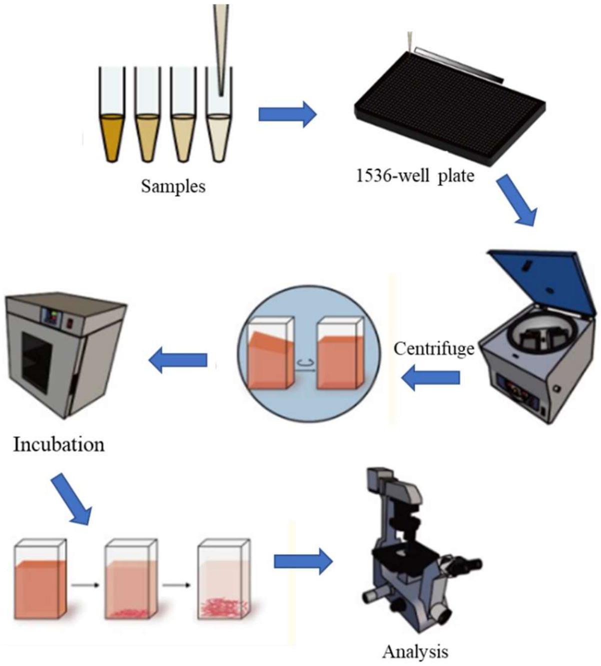

Figure S1. A flow diagram of the microliter-scale high-throughput screening system. Amyloid proteins are mixed with various concentration of inhibitors in a 1536-well plate. After incubation, they are imaged by fluorescence microscopy and the EC_{50} is calculated from fluorescence images.

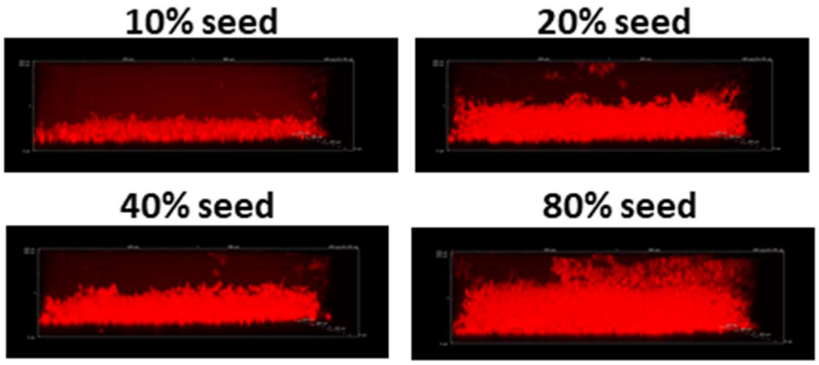

Figure S2. 10 μM α-synuclein aggregations with different seed concentrations. The percentage indicates the content of seed relative to the monomer concentration. 3D images were captured by a confocal microscope after incubation for 216 h.
